# Major depressive disorder prevalence and risk factors among Syrian asylum seekers in Greece

**DOI:** 10.1186/s12889-018-5822-x

**Published:** 2018-07-24

**Authors:** Danielle N. Poole, Bethany Hedt-Gauthier, Shirley Liao, Nathaniel A. Raymond, Till Bärnighausen

**Affiliations:** 1 Department of Global Health and Population, Harvard T.H. Chan School of Public Health, 665 Huntington Avenue, Boston, MA 02115 USA; 2 Signal Program, Harvard Humanitarian Initiative, Harvard T.H. Chan School of Public Health, 14 Story Street, Cambridge, MA 02138 USA; 3 Department of Biostatistics, Harvard T.H. Chan School of Public Health, 665 Huntington Avenue, Boston, MA 02115 USA; 4 Department of Global Health and Social Medicine, Harvard Medical School, 641 Huntington Avenue, Boston, MA 02115 USA; 5 Institute for Public Health, Faculty of Medicine, Heidelberg University, Im Neuenheimer Feld 130.3, 69120 Heidelberg, Germany; 6 Africa Health Research Institute, Mtubatuba, KwaZulu-Natal 3935 South Africa

**Keywords:** Migrants, Refugees, Global mental health, Conflict, Depression

## Abstract

**Background:**

Over one million Syrian asylum seekers have travelled to Greece with the ultimate aim of reaching other countries in western Europe. Depression prevalence and associated sociodemographic and displacement characteristics have been reported for resettled migrants. However, the prevalence of major depressive disorder (MDD) and its risk factors have not been described among migrants engaged in the asylum process ensuing from the Syrian crisis. This study provides new data about the mental health status of migrants in transition in the context of protracted asylum procedures.

**Methods:**

We conducted a cross-sectional survey in a Syrian refugee camp in the Attica region of Greece from January 16–31, 2017. Individuals ≥18 years of age with verbal Arabic or English language skills were eligible to participate. The Patient Health Questionnaire-8 (PHQ-8), an eight-item validated diagnostic and severity measure, was used to screen for MDD. We analysed the relationships between MDD and sociodemographic and displacement characteristics using multivariable logistic regression.

**Results:**

A total of 135 surveys were completed, representing 40% of the adult population in the refugee camp. The mean age of the participants was 30 years (18–61 years); women comprised 41% of the sample; 74% of the participants had ever married; 67% had children; and 33% of participants had not attended secondary school, including 11% who had never attended school. Median time since departing the country of origin was 12 months (< 1–74 months). Median time spent in the asylum process in Greece was 10 months (< 1–49 months). MDD was detected in 44% (95% CI: 37–50) of participants. Being a woman (Adjusted Odds Ratio [AOR]: 3.23, *p* = 0.019), each additional child (AOR: 1.61, *p* = 0.006), and increased time in the asylum process in Greece (AOR: 1.15, *p* = 0.043) were significant risk factors for MDD. Ever being married was associated with reduced odds of MDD (AOR: 0.23, *p* = 0.042).

**Conclusions:**

Syrian migrants face an extraordinarily high burden of MDD as they seek asylum. Incorporation of screening and treatment into service provision within refugee camps is urgently needed, particularly as migrants spend extended periods of time in transition due to protracted asylum procedures.

## Background

An estimated 4.5 million Syrians have sought asylum in neighboring countries since the start of the Syrian conflict in 2011 [1], including one million who have travelled to Greece with the ultimate aim of reaching other peaceful countries in western Europe [2]. Evidence suggests that the “healthy migrant effect” – the often-observed phenomenon that migrants are on average healthier than their host populations – does not apply to forced migrants fleeing conflict [3]. Instead, forced migrants may face higher rates of health problems, including mental health disorders [4].

Two previous studies found significantly higher rates of post-traumatic stress disorder (PTSD) among Syrian migrants compared to host populations [5, 6]. However, the emphasis on pre-migration trauma may overshadow other psychological needs of asylum seekers and the roles of displacement-related stressors [7, 8], which have been shown to be important determinants of mental health among Syrian refugees in Turkey [9]. Displacement-related stressors are known to contribute to the development of chronic mental health disorders, including anxiety [10] and major depressive disorders [11–13]. The EU-Turkey Statement of 2016, which assigned the responsibility of processing asylum claims to the first EU member state an asylum seeker entered, has likely contributed to extended asylum procedures for Syrian migrants in Greece and may subsequently have exacerbated the burden of mental health disorders [10]. Furthermore, several studies in post-conflict populations in the Middle East and Nepal have found that current living contexts strongly affect mental health [14]. A narrow focus on past trauma may result in failure to consider the effect of current life circumstances on mental health [15].

The development of depression during the asylum process is likely to undermine individual and societal functioning, which are essential for the survival and eventual resettlement of forced migrants [16]. Depression is also likely to lead to adverse acculturation outcomes [17].

While the early screening and treatment of depression may be expected to confer benefits, measures to detect or prevent psychological morbidity during the asylum-seeking phase of migration are only inconsistently implemented [18]. Previous studies reported estimates of the prevalence of depression among Syrians pre-conflict [19] and upon resettlement [20]. However, to date no study has measured the prevalence of depression among Syrians undergoing the protracted asylum process in refugee camps [21]. Furthermore, although sociodemographic characteristics (i.e. age, sex) and displacement-related stressors associated with depression and their moderating effects have been reported for resettled migrants [11], risk factors contributing to depression among Syrian asylum seekers have not been described.

In this study, we examined the prevalence and risk factors of major depressive disorder (MDD) among Syrians undergoing the asylum process in a European refugee camp. The purposes of the study are to 1) determine the prevalence of MDD among Syrian migrants living in a refugee camp in Greece using a validated measure, and 2) identify sociodemographic and displacement characteristics that could be risk factors for MDD. Quantifying the need for mental health services by estimating the prevalence of MDD and associated risk factors is the first step toward developing intervention programming for this population.

## Methods

This study included a face-to-face cross-sectional survey in a camp designated for Syrian refugees located in the Attica region of Greece. Data were collected from January 16–31, 2017. Access to the camp was granted by the International Organization for Migration (IOM), which managed camp operations.

### Sample size and sampling strategy

The sampling frame included all adults residing in the refugee camp during the study period (estimated 336 individuals). Individuals ≥18 years of age with verbal Arabic or English language skills were eligible to participate. A minimum representative sample of 97 participants was needed to estimate the prevalence of MDD with ±10% precision in the 95% confidence intervals (95% CIs).

**Fig. 1:**
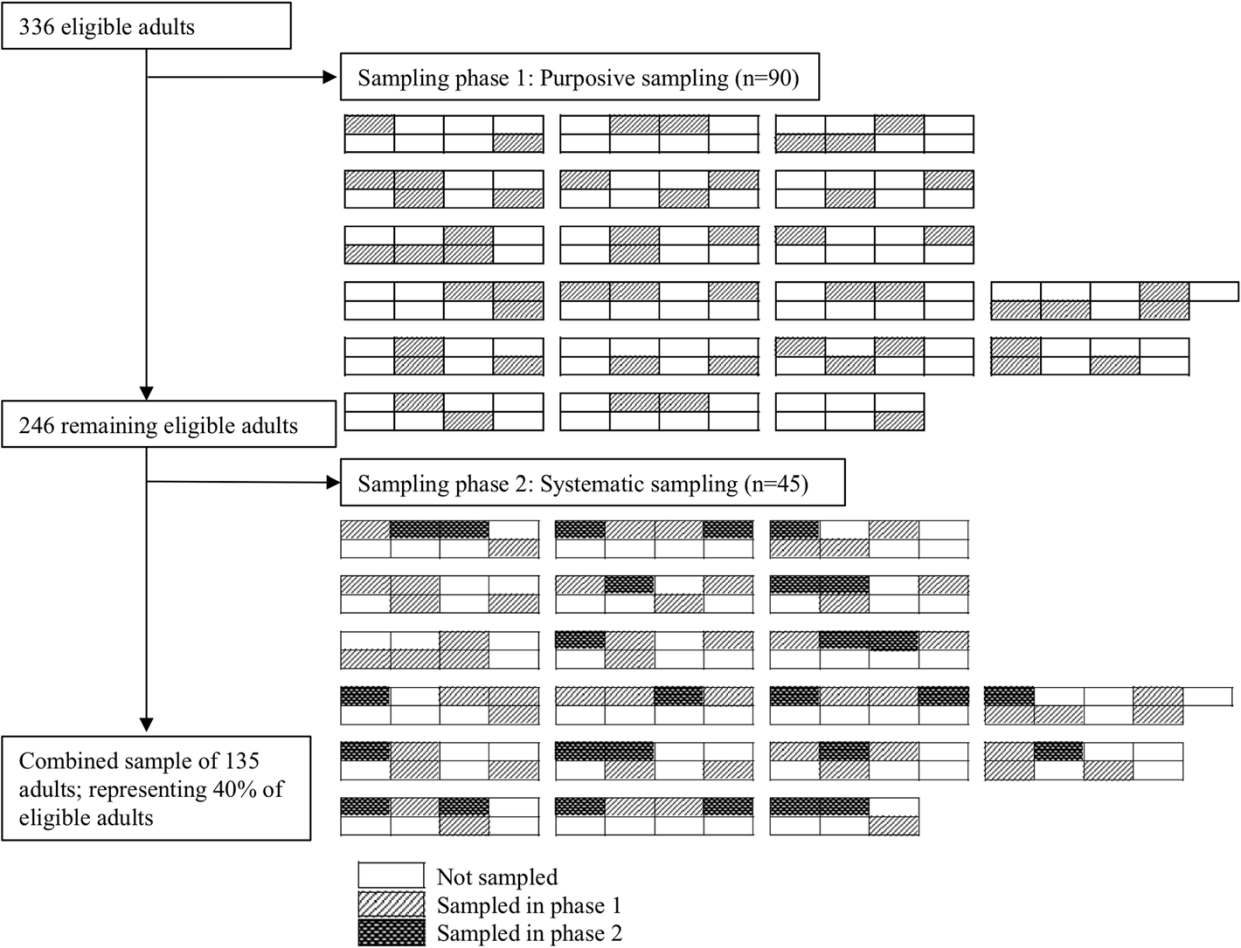
Sampling strategy

This study used mixed sampling procedures, utilizing a map of the camp to facilitate sampling. As shown in Fig. 1, camp inhabitants reside in containers, the primary housing unit. The containers provide housing for up to eight individuals, and are organized into blocks. In the first phase of sampling, due to the highly vulnerable nature of the population, camp management announced that a research study was being undertaken in the camp on the topic of migrant health. All eligible adults who volunteered to take part in the study were included (*n* = 90). No personally identifiable information was collected in order to protect the participants’ identities. The container numbers from which participants had been recruited were recorded to facilitate sampling in the second wave of recruitment. However, the container numbers were not linked to the survey data, precluding the identification of participants by container number.

After establishing familiarity with and receiving positive feedback regarding the research from camp management and the study population, a second container-based phase of sampling was deemed feasible and was performed to increase the representativeness of the sample. Briefly, a systematic sample was drawn using the camp map to recruit all eligible adults from exactly half the containers in each block. First, blocks in which less than half of the containers were represented during the first phase of sampling were identified and included in the second phase of sampling. All adults residing in the most north-western container of each block were recruited to participate in the study. Next, adults residing in neighbouring containers that were not previously sampled in the first phase were recruited, starting with the most western container and moving east. This process was repeated until all eligible adults from exactly half the containers in each block were recruited to participate in the study, producing an even distribution of sampled containers across the camp. Forty-five participants were included in the second phase of sampling, with a response rate of 92%. An illustration of the mixed sampling strategy is presented in Fig. 1. The total combined sample from the first and second phases of sampling was 135 participants.

### Procedures

All survey items were translated to and back-translated from Arabic. Face-to-face interviews took place in an area with audio privacy. There were several settings used to achieve this purpose, including a private area inside the participant’s container or outside on the veranda or in another outdoor area with audio privacy. The survey was administered by a member of the research team paired with Arabic-English interpreters. All members of the research team completed research training via the Collaborative Institutional Training Initiative and had experience working with migrant populations. The interpreters had professional experience working with Syrian refugee populations.

### Major depressive disorder

The primary outcome was the prevalence of MDD. MDD screening was conducted with the Patient Health Questionnaire-8 (PHQ-8) [22]. There is a lack of screening tools for depression severity specifically validated among Syrian refugees. However, the PHQ-8 has been used to assess MDD among migrant populations [23, 24], and has been validated in Arabic [25, 26]. A cut-off score of 10 or more was used for MDD on the basis of validation studies [22, 27]. The detection of MDD by the persistence and severity of depressive symptoms for two weeks [28] is an important threshold for clinical diagnostic assessments and treatment [29]. In our study, the PHQ-8 had a Cronbach’s α of 0.77, indicating satisfactory reliability of this scale among the study population.

Participants that reported depressive symptoms in the last two weeks were referred for assessment by an on-site psychologist employed by the IOM.

### Participant characteristics

Sociodemographic and displacement characteristics were collected using a survey. Level of education was used as a proxy measure of socioeconomic status in country of origin. The interview date, date of departure from country of origin, and date of arrival in Greece were used to calculate the total time displaced and time seeking asylum in Greece.

### Statistical analysis

The point prevalence of MDD was reported with Wald 95% CIs. Finite population correction was applied to adjust the prevalence estimate such that the variance applies only to the unsampled proportion of the population [30]. Missing data for items used to measure MDD status were replaced with minimum values, indicating the absence of depression, to produce the most conservative prevalence estimate. Sensitivity analyses were performed by imputing the maximum responses for observations with missing data for items used to assess depression status.

We addressed potential selection bias introduced by the mixed sampling methods in two ways. First, we assessed the balance of the sociodemographic characteristics (age and sex) of participants sampled in the first sampling phase with those sampled in the second phase. We used Welch’s approximation t-test to compare the age distribution of the study subsamples, accounting for heterogeneous variance due to differences in the sample sizes. Next, we used Pearson’s chi-square test to assess differences in the sex distributions of the study subsamples. We compared the prevalence of MDD between the sampling phases with Pearson’s chi-square test to evaluate selection bias on the outcome between the purposive and systematic samples.

Second, to determine whether the final combined sample (hereafter, the study sample) could be considered pragmatically random and representative of the sampling frame, we used camp census data collected during the study period to evaluate differences between the study sample and the camp population. Welch’s approximation t-test and Pearson’s chi-square test were used to compare the age and sex distributions of the study sample and the camp population, respectively. We additionally calculated the age- and sex- standardized MDD prevalence estimate using the population census data. After determining that the combined sample could be considered representative of the study population (Table 1), the total combined sample of 135 participants, representing 40% of the adult camp population, was used in the main analyses.

**Table 1:** Sample representativeness

Sample description		Age (years)				Percent women		
		N	Median	IQR	*p*-value ^†^	N	%	*p*-value^‡^
First and second sampling phases	Second phase	45	31	24–38		26	57.8	
	First phase	90	30	25–36	0.93	29	32.2	0.004
Combined sample and the camp population	Camp population	346	30	24–40		154	45.9	
	Combined sample	135	30	24–37	0.11	55	40.7	0.31

^†^Welch’s approximation t-test

^‡^Pearson’s chi-square test

We used descriptive statistics to quantify the participants’ sociodemographic and displacement characteristics. Associations between sociodemographic and displacement characteristics and MDD status were analysed by logistic regression and are reported as Unadjusted Odd Ratios (UORs) with 95% CIs.

We used multivariable regression to predict MDD with sociodemographic and displacement characteristics. Our model includes the following sociodemographic characteristics: gender, age, educational attainment, marital status, and number of children. Gender is included to account for established differences in depression prevalence between men and women [31]. Continuous age and age squared are included because depression prevalence has been shown to have a U-shaped distribution across the lifespan [32]. Educational attainment is included to account for the expected protective effect of increased education against depression [33]. Marital status, a known protective predictor of depression [34], is also included in the model. The number of children is included to capture the previously reported risk of depression associated with increased family size, which may exacerbate housing and financial pressures [35]. Total time since forced displacement represents time since exposure to conflict-related trauma, which has been shown to have an inverse relationship with the risk of depression [36]. Finally, time in the asylum process is included on the basis of literature demonstrating the deleterious effect of detainment and temporary protection on migrant mental health [13].

Adjusted Odds Ratios (AOR) with 95% CIs are reported for associations in the final multivariable logistic regression model. All analyses are complete case analyses. Statistical analyses were performed using Stata version 14.2 [37].

### Ethical considerations

This study was under ethics review, oversight, and governance both in Greece and in the United States by the IOM Greek research ethics advisory board and the Institutional Review Board of the Harvard T. H. Chan School of Public Health (Protocol IRB16–2015), respectively. Informed consent was obtained orally from all participants to avoid the potential risks of collecting participant names.

## Results

A total of 135 interviews were completed. As shown in Table 1, the age and sex distributions of the combined study sample did not significantly vary from those of the camp population at the time of the study (median age: 30 years for both, *p* < 0.11; and the percent women: 41 and 46%, respectively, *p* < 0.31). Therefore, for simplicity, we used the combined study sample in all analyses.

Participant sociodemographic and displacement characteristics are presented in Table 2. All (100%) participants had recently fled the Syrian conflict and the majority (89%) were of Syrian nationality. We did not record the nationality of those migrants who were not Syrian to maintain the anonymity of the collected data. The mean age of the participants was 30 years (range: 18–61 years); women comprised 41% of the sample; 74% of the participants had ever married; and 67% had children. 33% of participants had not attended secondary school, including 11% who had never attended any school. The median time since departing the country of origin was 12 months (< 1–74 months). The median time spent in the asylum process in Greece was 10 months (< 1–49 months).

**Table 2:** Participant sociodemographic and displacement characteristics

Participant characteristics	Total (*n* = 135)	Major depressive disorder (*n* = 59)
Nationality *n* (%)		
Syrian	120 (88.9%)	53 (89.8%)
Other	15 (11.1%)	6 (10.2%)
Gender *n* (%)		
Men	80 (59.3%)	27 (45.8%)
Women	55 (40.7%)	32 (54.2%)
Age median (years, IQR)	30 (24–37)	30 (22–39)
Education *n* (%)		
None	15 (11.4%)	8 (13.8%)
Any primary	29 (22.0%)	13 (22.4%)
Any secondary	38 (28.8%)	22 (37.9%)
Any tertiary or higher	50 (37.9%)	15 (25.9%)
Marital status *n* (%)		
Single, never married	34 (25.6%)	15 (25.9%)
Ever married	99 (74.4%)	43 (74.1%)
Number of children median (IQR)	2 (0–4)	2 (0–4)
Total time displaced (months) median (IQR)	12.1 (10.7–35.8)	12.0 (10.7–45.7)
Time in Greece (months) median (IQR)	10.4 (9.6–10.8)	10.4 (10.1–10.9)

MDD was detected in 44% (95% CI: 37–50) of participants. The prevalence of MDD did not significantly vary between the first and second sampling phases (43% vs. 44%, respectively, *p* = 0.902). The age- and sex-standardized MDD prevalence estimate was 46%.

Unadjusted and adjusted associations between sociodemographic and displacement characteristics and MDD status are presented in Table 3. Women had a significantly increased risk of MDD compared to men (UOR: 2.73, *p* = 0.005). Each additional child conferred significantly increased odds of MDD (UOR: 1.19, *p* = 0.040). MDD prevalence did not differ significantly by age, age squared, or educational attainment. Increased total time displaced (months) (UOR: 1.01, *p* = 0.179) and in the asylum process in Greece (months) (UOR: 1.10, *p* = 0.054) also exhibited trends toward increased odds of MDD.

**Table 3:** Associations between participant sociodemographic and displacement characteristics and major depressive disorder

Participant characteristics	UOR (95% CI)	*p*-value	AOR (95% CI) *n* = 120	*p*-value
Nationality				
Syrian	Ref		Ref	
Other	0.84 (0.28–2.52)	0.759	1.19 (0.31–4.55)	0.798
Gender				
Men	Ref		Ref	
Women	2.73 (1.34–5.55)	0.005	3.23 (1.21–8.62)	0.019
Age	1.00 (0.96–1.04)	0.898	0.82 (0.60–1.13)	0.224
Age squared	1.00 (1.00–1.00)	0.997	1.00 (1.00–1.00)	0.257
Education				
None	Ref		Ref	
Any primary	0.71 (0.20–2.48)	0.593	1.23 (0.24–6.43)	0.802
Any secondary	1.20 (0.36–4.00)	0.763	3.01 (0.59–15.45)	0.186
Any tertiary or higher	0.38 (0.12–1.22)	0.104	1.14 (0.21–6.17)	0.876
Marital status				
Single, never married	Ref		Ref	
Ever married	1.00 (0.44–2.13)	0.945	0.23 (0.05–0.95)	0.042
Number of children	1.19 (1.01–1.40)	0.040	1.61 (1.15–2.25)	0.006
Total time displaced (months)	1.01 (0.99–1.03)	0.179	1.01 (0.99–1.04)	0.355
Time in Greece (months)	1.10 (1.00–1.22)	0.054	1.15 (1.00–1.31)	0.043

**Fig. 2:**
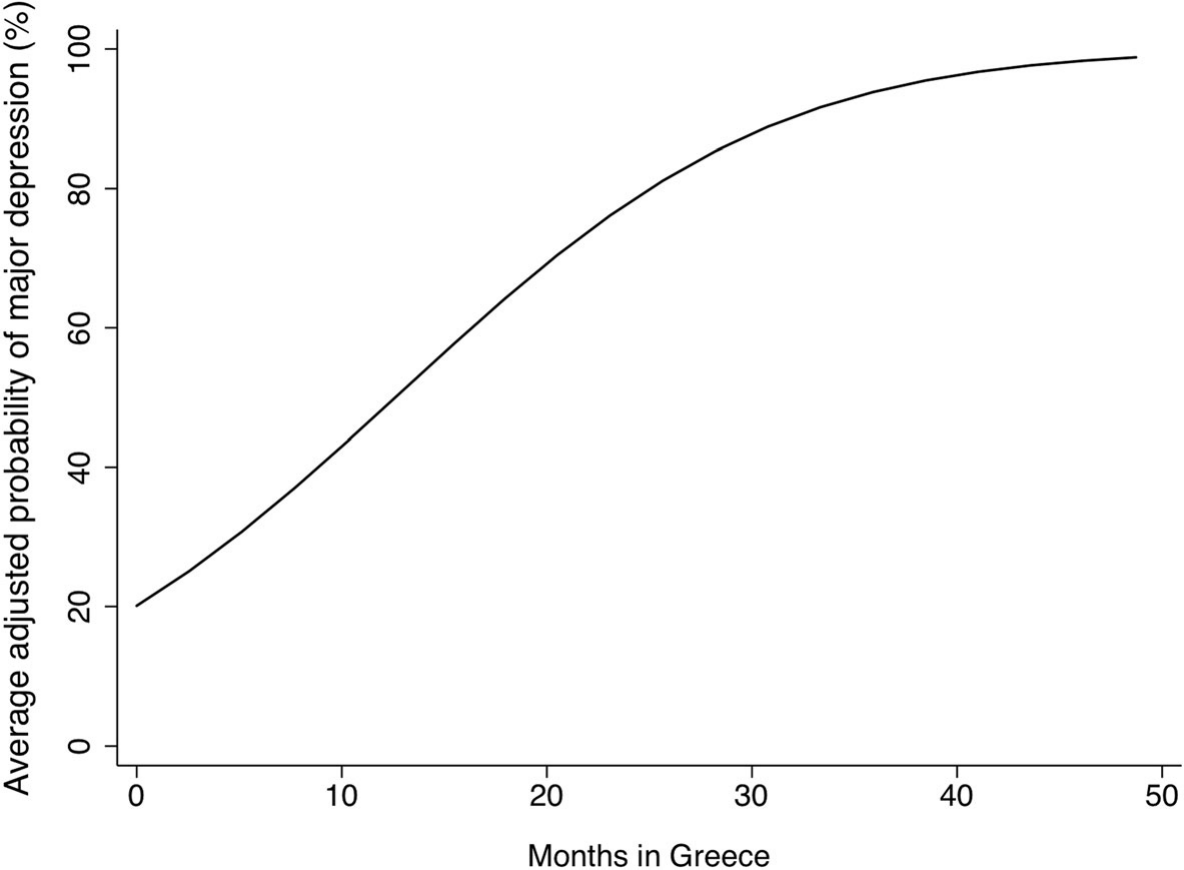
Major depressive disorder gradient with time seeking asylum. Average adjusted predictions at representative values of months in Greece

In the multivariable analyses, sociodemographic characteristics significantly associated with increased odds of MDD included being a woman (AOR: 3.23, *p* = 0.019) and each additional child (AOR: 1.61, *p* = 0.006). Marriage had a protective effect against MDD (AOR: 0.23, *p* = 0.043). Controlling for other factors, the odds of MDD increased 15% for each additional month spent in the asylum process in Greece (AOR: 1.15, *p* = 0.044). The average probabilities of MDD by months in Greece, while retaining the observed values for all other variables, are presented as percentages in Fig. 2. The inclusion of a dummy variable for sample phase in the adjusted model was not significant and did not qualitatively change the parameter estimates.

## Discussion

The prevalence of MDD was extremely high among Syrian asylum seekers in a refugee camp in Greece. This prevalence was nearly ten times higher than the depression prevalence among Syrians pre-conflict [19] and the global prevalence of depression [38]. The prevalence observed in this study was also nine times higher than the prevalence previously reported in a meta-analysis of studies among resettled refugees [4], and one and a half times higher than the prevalence reported among Syrian migrants in non-refugee camp settings in Jordan [20].

The high prevalence of MDD among Syrian asylum seekers supports the hypothesis that the “healthy migrant effect” may only apply to depression status when migration is motivated by economic reasons [39, 40]. Additionally, the very high prevalence of MDD among Syrian asylum seekers may be explained by the particular plight of fleeing conflict, the social and physical conditions in the refugee camp, as well as the protracted asylum process reported by the participants, a period extending beyond one year on average in Greece since the adoption of the EU-Turkey Statement [21]. Our finding that the prevalence of MDD increases with time in Greece supports the hypothesis that the protracted asylum process contributes to the development of depression among Syrian migrants. This finding is plausible, because migrants in refugee camps may lose hope for a better life as the transition phase waiting for asylum extends. In our study, the median time in the refugee camp was ten months. Previous studies have found reductions in depression with increased time since exposure to conflict [36]. Furthermore, while the study participants had been exposed to the Syrian conflict, assessing trauma as a risk factor for major depression was outside the scope of the present study. Our results are thus an important addition to the evidence base suggesting a countervailing time trend in depression prevalence if migrants are not quickly moved to resettlement and stable new life situations but instead remain in the uncertain situation of the asylum-seeking process in refugee camps.

MDD status varied significantly by several key sociodemographic characteristics. Women experienced a significantly higher burden of depression relative to men. Globally, the burden of depression is 50% higher among women [41], similar to the magnitude of the relationship between sex and MDD status observed in this study. The mental health literature includes extensive research about the physiological and psychosocial mechanisms that may explain the preponderance of depression among women [42]. Importantly, the effect of gender was independent of other sociodemographic and displacement characteristics in our study. A previous study among Syrian refugees in Jordan attributed the reported absence of a gender disparity in depression status as an artifact of equal exposure to trauma between men and women [20]; however, our results suggest the persistence of a gender disparity in the prevalence of depression.

Marriage conferred a protective effect against depression, consistent with the literature both globally and among Syrian refugees. The mechanism through which increased education reduces the odds of major depression may be the ability to access social support and resources, which may be dependent on marital status.

Our study contributes to the existing evidence on migrant mental health by showing evidence of significantly increased odds of major depression with each additional child. This finding provides further motivation for the urgency of screening and treatment of depression among asylum seekers, as parental depression and PTSD are significantly associated with negative and withdrawn parenting and adverse child physical and psychosocial health [43, 44], while the remission of parental depression predicts improved functioning in their children [45].

Our findings have several important policy implications. First, the overall high prevalence of MDD in this refugee camp motivates the need for consistent screening and treatment. Second, the magnitude of the gender disparity in the global prevalence of depression was also observed among our study population of Syrian migrants. Epidemiological research of depression among migrants, and subsequent intervention design and implementation, should be responsive to the particular situations faced by women in refugee camps. Third, familial characteristics such as marital status and number of children may be important protective and risk factors, respectively, for the development of depression.

Time spent in the refugee camp is a significant, and modifiable, risk factor for MDD. In light of the high prevalence of MDD, even upon arrival in camp, screening and treatment are necessary both at arrival and throughout the duration of time spent in camp in order to identify baseline and incident depression. Implementation research can contribute to reducing the burden of depression among asylum seekers by identifying best practices in relation to screening and treatment strategies. Such interventions must be responsive to the socioeconomic factors associated with depression, particularly so as not to exacerbate gender disparities or the risk posed by larger families. Ultimately, reducing the duration of time spent in the asylum process, both in camps and in reception centers, and thus the adverse effect of detainment and temporary protection on depression, should be prioritized.

Our study has several limitations. First, selection on depression status itself as well as on other participant characteristics may have biased the prevalence estimate. Biases resulting from selection on the outcome, depression, can theoretically be addressed using Heckman-type selection models. For this purpose, we have developed variants of Heckman-type selection models in other survey applications, using the identity of the interviewer as a selection variable [46–49]. In this study, however, we did not employ a sufficiently large number of interviewers to be able to exploit variation in interviewer identity for Heckman-type selection models. In future larger survey studies on depression among migrants, we will have the opportunity to apply Heckman-type selection models to correct for potential selection on depression. Additionally, selection on other participant characteristics may have biased our results. Comparisons of the final combined sample with camp census data suggest our sample was representative of the camp adult population age and sex distributions, and the age- and sex-standardized prevalence estimate was similar to the sample prevalence estimate, providing further evidence of the representativeness of our sample. Nonetheless, comparability of measured characteristics does not exclude the potential for bias resulting from unmeasured characteristics, e.g., general propensity to engage and extraversion (which are likely lower among people with depression).

Second, while our findings suggest potential risk and protective factors of major depression among migrants, the cross-sectional nature of our study precludes causal inference. Future prospective designs could increase the strength of causal inferences regarding the effects of sociodemographic and displacement characteristics. Randomized controlled intervention studies could strengthen the evidence-base of the effectiveness of psychological treatments, including problem-solving counselling, interpersonal therapy, cognitive behavioral therapy, and behavioral activation, administered by community health workers or via mHealth platforms [50–53].

Finally, the external generalizability of our findings may be limited by camp-level characteristics specific to our study settings. However, other refugee camps in mainland Greece, under the jurisdiction of Greece’s Ministry of Migration, have similar observable characteristics.

## Conclusions

Syrian migrants face an extraordinarily high burden of MDD during the asylum process. Time spent in camps is a significant risk factor for MDD that can be immediately alleviated by expediting resettlement. Incorporation of depression screening and treatment into service provision within refugee camps is urgently needed to mitigate the effects of time in camps as governments work to address the protracted asylum process.

## Abbreviations

AOR: Adjusted odds ratio
IOM: International Organization for Migration
MDD: Major depressive disorder
PHQ-8: Patient Health Questionnaire-8 item
PTSD: Post-traumatic stress disorder
UOR: Unadjusted odds ratio
